# The time course of metabolic plasticity and its consequences for growth performance under variable food supply in the northern pike

**DOI:** 10.1098/rspb.2022.0427

**Published:** 2022-05-25

**Authors:** Viktor Nilsson-Örtman, Christer Brönmark

**Affiliations:** Department of Biology, Aquatic Ecology Unit, Lund University, Ecology Building, 22362 Lund, Sweden

**Keywords:** time course, performance, metabolic plasticity, growth, feeding

## Abstract

Many species up- or downregulate their resting metabolic rate (RMR) when they encounter favourable or unfavourable feeding conditions, respectively. This is thought to promote faster growth when food is abundant and conserve energy reserves when food is scarce. The time it takes to express metabolic plasticity remain little studied. Here, we develop a conceptual model showing how rapid or slow metabolic plasticity alter growth trajectories in response to changes in food supply. We test predictions from the model in a food manipulation experiment with young-of-the-year northern pike, *Esox lucius*, a species that experience drastic changes in food supply in nature. We find that metabolic plasticity is expressed gradually over several weeks in this species. Rapid changes in food supply thus caused apparent trait-environment mismatches that persisted for at least five weeks. Contrary to predictions, pike grew faster at high food levels when they had previously experienced low food levels and downregulated their RMR. This was not owing to increases in food intake but probably reflected that low RMRs increased the energetic scope for growth when feeding conditions improved. This highlights the important but complex effects of metabolic plasticity on growth dynamics under variable resource levels on ecologically relevant time scales.

## 1. Introduction

Most organisms experience considerable variation in environmental conditions over the course of their life. To cope with this variability, many species have evolved the ability to adaptively adjust their physiology, morphology and behaviour in response to environmental change [1–4]. This form of reversible phenotypic plasticity can have important consequences for individual fitness and the outcome of ecological interactions in variable environments [5–7] and promote resilience to climate change [8].

There is an increasing awareness that not only the magnitude but also the rate at which plasticity is expressed can be important for organismal performance and ecological interactions [6,9–12]. According to theory, the fitness advantage an organism can gain from being plastic will depend on how rapidly a fitness-related trait can change over time, how much it can change, and the costs associated with being plastic, relative to the rate, magnitude and predictability of environmental change [13,14]. Plasticity can thus increase fitness substantially if it occurs rapidly and the environment changes slowly and predictably. However, if plasticity is slow and the environment changes fast, this can lead to persistent mismatches between an individual's phenotype and its environment that cause costs of plasticity to outweigh its benefits [7].

Plasticity in resting metabolism in response to changes in food supply may be especially relevant in this context. Experiments on a wide range of organisms have shown that resting metabolic rates (RMR; the respiration rate of non-digesting, non-reproducing organisms at rest) tend to increase when food is abundant, and decrease when food is scarce [15–17]. The strength of these plastic responses differ between species [15,16,18,19] and some species harbour considerable variability in the strength of metabolic plasticity among individuals [1,20]. Perhaps the most promising explanation for these observations is provided by the ‘context dependence hypothesis’, which posits that high-RMR phenotypes have high fitness under favourable conditions, whereas low-RMR phenotypes have high fitness under poor conditions, which will favour metabolic plasticity when individuals experience fluctuations in feeding conditions over the course of their lives [20]. Some studies of intraspecific variation in metabolic plasticity lend support to this idea, showing that individuals that downregulate their RMR the most at low food levels tend to loose mass and fat reserves at a slower rate, and have higher survival, when food is scarce [1,21], while individuals that upregulate their RMR the most at high food levels tend to grow faster when food is abundant [1,21–23]. The latter presumably arising because high-RMR phenotypes have a greater capacity to acquire, process or convert food into biomass than low-RMR phenotypes that more than compensates for the higher energetic demand associated with increases in RMR.

Few studies have quantified how rapidly metabolic plasticity is expressed in response to changes in food availability, but the available data suggests that it is a relatively slow process that occurs on a time scale of one to several weeks in fishes [24–27]. This is consistent with the fact that plastic changes in many similarly complex physiological and morphological traits require several days, weeks or even months to be fully expressed [28–34], although some traits can be altered substantially over minutes or hours [35,36]. Based on this, it is not surprising that particularly strong metabolic plasticity has been observed in response to slow and predictable fluctuations in temperature and food availability that occur over seasonal time scales [37–39]. However, a wide range of environmental factors can change on a shorter time scale which may cause the expression of metabolic plasticity to be rate-limited. Weather events may cause shifts from favourable to unfavourable conditions over a period of days to weeks, and the phenological timing of key events in interacting species can drastically alter feeding conditions from one week to the next [40]. The extent to which organisms respond to environmental change at these time scales through metabolic plasticity remains poorly understood.

Here, we explore the rate and time course of metabolic plasticity, and its effect on growth performance, in response to short-term changes in food supply in the northern pike, *Esox lucius* Linneaus 1758. In Lake Krankesjön, southern Sweden, this species experience rapid fluctuations in food supply over the course of a few weeks triggered by the seasonal migration of its main prey species, roach, *Rutilus rutilus* [41,42]. Pike may thus benefit from being able to rapidly and reversibly adjust its basal metabolic requirements in response to relatively rapid changes in food supply that occurs on a time scale of days to weeks.

**Figure 1.**
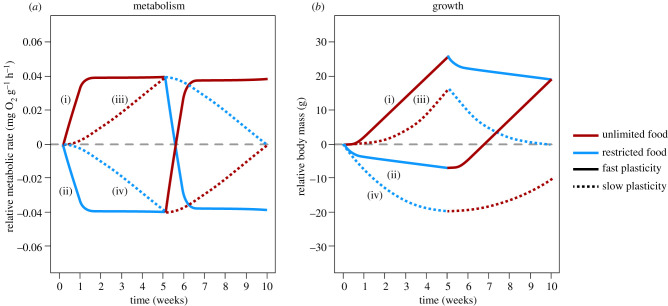
A conceptual model showing how the rate of metabolic plasticity influences growth performance in response to changes in food supply under the context-dependence hypothesis. This hypothesis posits that resting metabolic rates (RMR) will increase at high food levels and decrease at low food levels; and that high RMR will promote faster growth at high food levels, but cause faster mass loss at low food levels. (*a*) Predicted changes in RMR over time when switched between high and low food levels; (*b*) predicted changes in body mass over time. Solid lines show predictions for an organism with rapid metabolic plasticity switched from high-to-low (i) or low-to-high (ii) food levels. Dashed lines shown predictions for an organism with slow metabolic plasticity switched from high-to-low (iii) or low-to-high (iv) food levels. (Online version in colour.)

We first present a conceptual model showing how metabolic plasticity can alter growth trajectories in response to changes in food supply under the context-dependence hypothesis (figure 1). The conceptual model is based on the following assumptions. First, we assume that pike will upregulate their RMR at high food levels, and downregulate their RMR at low food levels until reaching an upper and lower limit, respectively (figure 1*a*). For simplicity, we assume that up- and downregulation occur at the same rate, but allow this rate to vary. Second, we assume that high RMRs enable individuals to grow faster at high food levels, but lose mass faster at low food levels [20]. Third, we assume that growth is linear for a given combination of food supply and metabolic rate so that any curvature in growth trajectories arise as a result of metabolic plasticity (i.e. nonlinear growth occurs when RMR changes over time; linear growth occurs when RMR is constant). Finally, we assume that individuals express intermediate RMRs at the onset of the time period considered in the model.

Based on this conceptual model, we ask how pike with rapid (days) or slow (weeks) metabolic plasticity will change in mass when switched from high to low (figure 1, red-to-blue) or low to high (figure 1, blue-to-red) food levels. After each change in food levels, we expect individuals to experience a period of phenotype-environment mismatch in resting metabolism. Thus, when switched to low food levels, individuals will initially express higher than optimal RMRs, and, because of this, will rapidly lose body mass immediately after the switch (figure 1*b*, blue growth trajectories). By contrast, when switched to high food levels, individuals will initially express lower than optimal RMRs, and grow slowly immediately after the switch, despite the high abundance of food (figure 1*b*, red growth trajectories). After some time, we expect metabolic plasticity to reduce or eliminate this phenotype-environment mismatch. If metabolic plasticity is very rapid, as, for example, if a species can reach its minimum or maximum resting metabolism within a week, we expect that this mismatch phase will be brief. As a result, growth performance will increase rapidly over time and stabilize at a constant high level (slow mass loss at low food levels, rapid growth at high food levels) as RMRs reach the upper and lower limit, respectively (figure 1, solid trajectories). On the other hand, if metabolic plasticity is slow, so that it takes many weeks for individuals to reach their minimum or maximum RMR, the duration of the mismatch period will span the entire time period. As a consequence, we expect growth to be slower overall, and show a slow gradual increase/decrease over the entire period (figure 1*b*, dashed growth trajectories).

To test these predictions, we performed an experiment where we acclimated young-of-the-year pike from lake Krankesjon to laboratory conditions for four weeks, and then abruptly switched them to high or low food levels, which they experienced for five weeks, before being switched to the opposite conditions for five weeks. We monitored changes in RMR and growth every week to quantify the rate and time course of metabolic plasticity, and its effect on growth performance, which we expect to be strongly and positively correlated with fitness in this species.

## 2. Methods

### (a) Rearing conditions

Young of the year (0+) pike (*E. lucius*) were caught by electrofishing in Lake Krankesjön (55°42'28″ N, 13°28'24″ E) in southern Sweden and brought to the laboratory in Lund (*n* = 18, mean wet weight ± 1 s.d. = 109 ± 20 g, range = 66 g to 141 g). Nine individuals were caught on 27 September 2018, and nine individuals were caught on 7 November 2018. Except for the start date, both groups were treated identically. Pike were acclimated for four weeks before the food manipulation experiment started. During the acclimation period, individuals were starved for one week, then accustomed to eating live prey by occasional feeding (two to six small prey fish in total) for two weeks. During the fourth week of the acclimation period, all individuals were fed exactly once, three days before the experiment started. Pike were maintained in individual aquaria (95 × 44 × 40 cm) with recirculated and filtered water at 18.2 ± 0.6°C (mean ± 1 s.d.) and a constant 12 h : 12 h light : dark photoperiod. Aquaria were covered with black adhesive paper on three sides and contained numerous artificial plants for habitat structure and prey shelter. The study was performed under permission from the Malmö/Lund authority for ethics of animal experimentation (Licence M36-14).

### (b) Food manipulations

Each individual pike was randomly assigned to one of two treatments: high-low (HL: five weeks at high food levels, followed by five weeks at low food levels); or low-high (LH: five weeks at low food levels, followed by five weeks at high food levels). We originally assigned nine individuals to each treatment, but two individuals escaped, both belonging to the HL treatment, producing a final sample size of 16 individuals (seven HL, nine LH). Wild-caught crucian carp (*Carassius carassius*) were used as food throughout the experiment (mean wet weight ± 1 s.d. = 6.7 ± 3.3 g; range 1.6 g to 21.6 g). At high food levels, pike received four crucian carp per week (one per day on Tueday, Wednesday, Thursday and Friday). At low food levels, pike received one crucian carp per week (typically Tuesday, see below). Owing to an error, pike were only fed three times on week 6 in the high food treatment. Prey fish were chosen haphazardly on each feeding event. Mean prey size did not differ among individual pike (Anova, *F*_15,528_ = 0.74, *p* = 0.74). On average, pike received 6.6% of their wet weight in food per week in the low food treatment and 23.4% of their weight per week in the high food treatment. Prey fish not eaten within 24 h were removed, but 96% of prey fish was consumed.

### (c) Measuring resting metabolic rate and growth

Each week, we measured the wet weight and resting metabolic rate (oxygen consumption rate, RMR) of each individual. Individual wet weight was measured at the end of each metabolic rate assay by gently transferring pike to a container of water on a digital scale. We estimated RMR using intermittent flow closed system respirometry. To ensure that individuals were in a post-absorptive state, we performed metabolic rate assays on Mondays and fed the pike on Tuesdays-Fridays. For practical reasons, we occasionally performed RMR essays a day before or after (i.e. Sunday or Tuesday), but they were always performed at least 48 h after feeding. The respirometer chamber was custom built from Perspex, measured 80 × 90 × 300 mm and contained 3.1 l water, including the inlet and outlet tubing [43]. A magnetic stirrer ensured adequate mixing. A Oxyguard Handy Polaris 2 oxygen meter probe (OxyGuard A/S, Farum, Denmark) was inserted into the chamber through a hole sealed with a rubber gasket and oxygen levels were recorded at 10 or 30 s intervals. The water temperature in the respirometer was maintained at 18.4°C ± 0.15°C (mean ± 1 s.d.). At the beginning of each RMR assay, an individual pike was gently transferred to the respirometer with a net, the chamber was closed, and continuously flushed with oxygen-saturated water for 5 min to allow the individual to settle. After 5 min, we turned off the pump and closed both the inlet and outlet valves. The chamber was carefully checked for leaks and any bubbles removed when the chamber was closed. Assays were terminated when the oxygen concentration reached approximately 80% and typically lasted between 10 to 20 min in total. We discarded measurements from the first 2 min after closing the valves to minimize short-term effects of handling stress. Nearly all fish remained motionless throughout the assays. However, as fish spent a relatively short amount of time in the respirometer before measurements began (7 min), our metabolic rate measurements are likely to be affected by handling stress to some extent. Consequently, we will refer to our estimates as resting metabolism, rather than standard metabolism [44]. We favoured this approach to keep the total assay time as brief as possible, as we wanted to assay each individual repeatedly each week throughout the experiment. However, all individuals settled down in the respirometer very quickly and showed no visible signs of stress (e.g. no burst swimming or rapid gill movements) a few seconds after closing the respirometer. We are thus confident that the effect of handling is relatively minor, and emphasize that our aim is to assess changes in the magnitude of metabolic rates over time, rather than provide estimates of absolute metabolic rate levels in this species (for a discussion, see [45]).

We estimated the rate of oxygen uptake (mg O_2_ h^−1^) using linear regression, and calculated the mass-specific metabolic rate (MO_2_) as: MO_2_ = [(*V*_r_ – *V*_f_) x ΔO_2_]/M_f_, where *V*_r_ is respirometer volume, *V*_f_ is fish volume (assuming that pike have the same density as water), ΔO_2_ is the rate of oxygen uptake and M_f_ is fish mass [44]. Fish volume ranged from 2.5% to 5.5% of the total volume of the respirometer. We cleaned the respirometer and holding tanks with 75% ethanol at the end of each session and monitored background respiration levels in empty controls at least once per day. Background respiration rates were found to be negligible (0.00274 ± 0.009 mgO_2_ h^−1^) and constant over time; consequently, they were ignored. No temperature correction was performed owing to the small temperature range. All essays were performed between 08.30 and 17.00 to minimize the effect of diurnal changes in metabolism. Individuals were essayed in a random order on each day.

### (d) Analysis

For each individual fish, we had measurements of its resting metabolism (RMR; mg O_2_ g^−1^ h^−1^) and mass (wet weight, in g) taken at 11 points in time over the course of the experiment. Three RMR measurements were identified as outliers using the *tsoutlier* function in the *forecast* R package [46]. We replaced these observations using linear interpolation by applying the *tsclean* function from the same package to individual-level time-series data. Subsequent analyses did not change qualitatively depending on whether these observations were replaced or removed. Because observations on RMR and mass were unequally distributed in time, we analysed the results using mixed-effects models appropriate for such data [47]. We tested for the effects of food level (high and low food level), time in treatment (number of days after a food treatment switch; continuous), treatment group (HL and LH) and their interactions on RMR and log_10_-transformed wet weight separately using repeated measure linear mixed models (LMM) using the *lme* function in the *nlme* R package [48]. Wet mass was included as a covariate in the RMR model to control for allometric effects. To account for the potential temporal autocorrelation within individuals, we modelled individual identity as a random effect, and modelled the residual covariance structure as an autoregressive model of order 1, where the temporal autocorrelation is strongest for observations from adjacent time periods within each individual.

To further explore the shape of metabolic trajectories and their consequences for growth performance, we modelled changes in RMR and log_10_-transformed wet mass (g) as a nonlinear function of time (days since start of the experiment) using generalized additive models (GAM) and generalized additive mixed models (GAMM). These are non-parametric extensions of generalized linear (mixed) models that require no *a priori* assumptions about the functional form of the modelled relationship. To test for differences between groups in the shape of metabolic trajectories, we fitted GAM models of the form: *Y_i_*_,*j*_ = α + *f*_1_(*x*_1*i*,*j*_) + *f*_2_(*x*_2*i*,*j*_) × *x*_3*i*,*j*_ + *β_i_* + *ε_i_*_,*j*_, where *Y_i_*_,*j*_ is the response variable (metabolic rate or wet mass) of individual *i* in week *j*, α is the intercept, *f*_1_(*x_i_*) is a smoothing function for the first food treatment (HL), *f*_2_(*x*_2*i*_) is a smoothing function for the second food treatment (LH), *X*_3*i,j*_ is 0 for individuals in the first food treatment and 1 otherwise, *β_i_* is an intercept for each individual, and *ε_i_*_,*j*_ are residuals. Models were fitted using a normal error distribution and an identity link function. Optimal parameters of the smoothing functions were estimated based on generalized cross-validation scores that balance goodness-of-fit against model complexity. We tested for significant differences between groups based on Akaike information criterion (AIC) scores and *F*-tests between models with and without separate smoothing functions for each treatment. Because the shape of the trajectories differed, we estimated group-level trajectories using two separate GAMM models of the form *Y_i_*_,*j*_ = α + *f*_1_(*x*_1*i*,*j*_) + *b_i_* + *ε_i_*_,*j*_ using data from each group (HL and LH), with notation as above, but modelling individual intercepts as a random effect *b_i_*. We present predicted values and prediction standard errors from these GAMM models graphically. All statistical analyses were performed in R v. 4.0.0 [49].

## 3. Results

**Figure 2.**
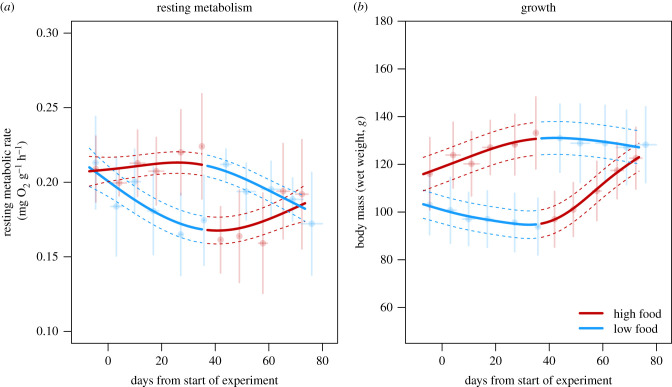
Changes in resting metabolic rate (*a*) and wet weight (*b*) in young-of-the-year pike when switched between limiting food levels (blue; fed once per week) and satiating food levels (red; fed four times per week). The plots show group means trait values ±95% CIs for each measurement event (points and vertical error bars). Horizontal error bars show ±95% CIs for the mean age at each measurement event. Nonlinear trajectories represent our best-fitting predictions for the shape of the underlying trajectories estimated using generalized additive mixed models. (Online version in colour.)

The RMR of young-of-the-year pike changed dynamically over time when individuals were switched from high to low (HL) and low to high (LH) food levels (figure 2*a*). The mean RMR did not differ between groups on the first measurement event (Welch's *t*_13.53_= −0.22, *p* = 0.83) with a mean RMR ±95% confidence interval (CI) of 0.209 ± 0.028 mg O_2_ g^−1^ h^−1^ in the HL group and 0.213 ± 0.036 in the LH group. The food treatments (high or low food) had a significant effect on how RMRs changed with time after being switched (days after food switch × food level interaction; *F*_1,153_ = 15.45, *p* < 0.001). RMRs increased and decreased at a similar rate at a given food level in both groups (LMM; days × food level × group interaction; *F*_1,153_ = 1.25, *p* = 0.264). RMRs increased at a rate of 0.0038 ± 0.0027 mg O_2_ g^−1^ h^−1^ per week at high food levels, and decreased at a rate of −0.008 ± 0.002 mg O_2_ g^−1^ h^−1^ per week at low food levels (mean ± 1 s.d.). This approximately represents a 9% increase in RMR after five weeks at high food levels and a 18% decrease in RMR after five weeks at low food levels. As a result, the two groups first diverged in RMR and differed significantly in RMR on measurement events 5 and 7 (Welch's *t*-test; both *p* < 0.02), and close to significantly on week 6 (Welch's *t*-test; all *p* = 0.057) and then converged in RMR after being switched to opposite food levels (figure 2*a*). At the time of being switched to opposite conditions, the mean RMR ±95% CI was 0.241 ± 0.063 mg O_2_ g^−1^ h^−1^ in the HL group, and 0.171 ± 0.078 mg O_2_ g^−1^ h^−1^ in the LH group (Welch's *t*_10.825_ = 3.722, *p* = 0.003. At the end of the experiment, on week 12, the two groups did not differ in mean RMR (*t*_3.308_= 0.14, *p* = 0.90) with a mean RMR ±95% CI of 0.190 ± 0.074 in the HL group and 0.199 ± 0.062 in the LH group. Fish mass had no effect on RMR (*F*_1,153_ = 0.518, *p* = 0.47), indicating weak allometric effects, which can be expected given the limited size range studied.

Changes in wet weights over time were similarly dynamic (figure 2*b*). Fish in the HL treatment tended to be somewhat heavier than fish in the LH treatment at the start of the experiment, with a mean start weight ±95% CI of 116 ± 20 g in the HL group and 103 ± 19 g in the LH group, although this difference was not statistically significantly (*t*_13.4_= 1.23, *p* = 0.24). As expected, the food treatments had a significant effect on growth (days × food level interaction; *F*_1,154_ = 246.0, *p* < 0.001) as fish gained weight at high food levels, and lost weight over time at low food levels as expected. The rate at which fish lost or gained mass at a given food level differed between HL and LH groups (days × food levels × group interaction; *F*_1,154_ = 20.2, *p* < 0.001). This reflected that fish grew faster at high food levels in the LH group than in the HL group.

The nonlinear generalized additive models on RMR as a function of time confirmed that the two groups differed in the shape of their metabolic trajectories (GAM models with and without separate smoothing functions for each group; ΔAIC = −11.1; *F*_5.7,153_ = 3.24, *p* = 0.006). Prediction from group-specific GAMM models provided further information on the shape of the trajectories of metabolic plasticity (figure 2*a*; nonlinear trajectories). In the HL group, RMRs increased at a relatively slow rate in the high food treatment and reached a maximum before being switched to low food levels, indicating limited scope for further increases in RMR or, possibly, a relatively substantial time-lag in the upregulation of RMR. After being switched to low food, RMRs began to decrease linearly almost immediately, indicating little or no time lag in the downregulation of RMR. In the LH group, RMRs decreased near-linearly from the beginning of the experiment, reaching a minimum after the switch to high food levels, followed by a slower, near-linear increase in RMR at high food levels (figure 2*a*). Again, the results indicate little or no time lag in the downregulation of RMRs, but a possible tendency for a time-lag in the upregulation of RMRs.

The GAM/GAMM models on wet mass revealed the shape of growth trajectories in each group. As expected, growth trajectories differed significantly between treatments (GAM models on log-transformed wet weights with and without separate smoothing functions for each treatment: ΔAIC = 159.7; *F*_3.9,150_ = 60.35, *p* < 0.001). Predictions from the group-specific GAMM models on untransformed wet weights showed that, in the HL treatment, individuals gained in wet mass at an even, moderate rate at high food levels, and decreased in wet mass at an even but slower rate after being switched to low food levels after six weeks (figure 2*b*). In the LH treatment, pike lost wet mass at a slow, even rate at low food levels, but gained wet mass at a rapid, even rate after being switched to high food levels (figure 2*b*). In all cases, the increase and decrease in wet mass were very close to linear, showing little curvature indicative of changes in growth performance owing to gradual up- or downregulation of RMR.

**Figure 3.**
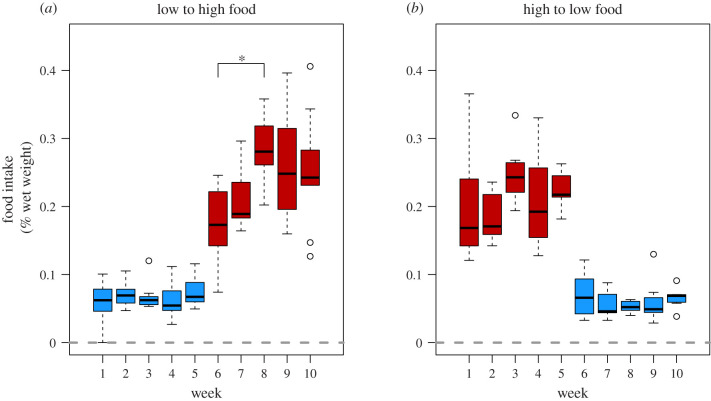
Weekly food intake rates in young-of-the-year pike switched from low to high food levels (*a*) or high to low food levels (*b*). Note that all fish were fed once the week before the experiment started (week 0, not shown). (Online version in colour.)

Pike consumed on average 21.3% of their wet weight in food per week in the high food treatment, and 6.3% of their wet weight in food per week in the low food treatment (figure 3). The weekly food intake remained constant over time within each feeding regime (Bonferroni-corrected pairwise *t*-test; *p* > 0.05) with the exception that food intake was lower on week 6 than on week 8 in the high food treatment (Bonferroni-corrected pairwise *t*-test; *p* = 0.021). This, however, reflected that pike were only fed three times (not four) on week 6 in the high food treatment.

## 4. Discussion

Young-of-the-year pike showed considerable phenotypic plasticity in their RMRs in response to rapid changes in food availability. Plastic changes in RMRs were reversible but required at least five weeks to be fully expressed. This shows that metabolic plasticity can be sufficiently fast to produce meaningful changes in RMR in response to relatively short-term changes in food availability, such as when a large proportion of a species’ dominant prey migrate over a period of a few weeks, but sufficiently slow to be rate-limited following rapid environmental change, causing trait-environment mismatches that can persist for several weeks and cause reductions in growth performance and body condition. These results highlight the need to incorporate information on the time course of metabolic plasticity for understanding predator growth strategies in response to variable feeding opportunities [9,12].

Pike downregulated their RMR more than 1.8 times faster than they upregulated their RMR. Trajectories for the downregulation of RMR was strikingly consistent in the two groups, and RMRs began to decline nearly immediately when the HL group was switched to low food levels. This suggests that downregulation of RMR occurs rapidly and with little or no time lag in pike. By contrast, upregulation of RMRs was decidedly slower in both groups. Perhaps the most parsimonious explanation for this result is that upregulation of RMRs was subject to a substantial time-lag; i.e. that pike had to experience high food levels for several weeks before they responded by increasing their RMR. Theory suggests that such a time lag can arise either owing to time constraints on information acquisition or trait expression [7,14]. Based on group-by-food treatment means, RMRs did appear to remain low for a few weeks at high food levels in both groups before they began to increase; but it should be noted that the more robust test provided by the GAMM analysis did not find statistical support for any upward curvature in the metabolic trajectories, which would be expected in the presence of a significant time lag. Further studies with a higher temporal resolution will be needed to elucidate these mechanisms in greater detail.

The fact that RMRs reached their maximum level in the HL group before individuals were switched to low food levels raises the intriguing possibility that pike in this group approached an upper limit of metabolic plasticity. This would indicate that the pike studied here were operating at close to their maximum metabolic capacity at the beginning of the experiment. If this is representative of pike in lake Krankesjön in autumn, which may represent a distinct peak in pike feeding opportunities, it is worth exploring further using field-based respirometry.

The observation that pike increased their RMR at high food levels and decreased their RMR at low food levels is consistent with predictions from the context dependence hypothesis [20]. However, contrary to predictions, the upregulation of RMRs at high food levels did not promote faster growth. Instead, pike grew significantly faster at high food levels when they had first been starved for five weeks and, consequently, had downregulated their RMRs (figure 2*b*). This growth acceleration represents an example of compensatory growth—an increase in growth rates that occurs in many organisms when favourable conditions are restored after a period of adverse growth conditions [50–52]. Most cases of compensatory growth in fishes have been attributed to hyperphagi, where previously starved fishes exhibit significantly more elevated feeding rates than fishes which have not experienced food reductions [50]. This, however, was not the case here, as pike in the LH group did not consume significantly more food at high food levels than pike in the HL group (figure 3). Alternatively, it has been argued that a reduction of metabolic rates during starvation may increase growth efficiency when beneficial feeding conditions are restored [50,53]. We suggest that this represents the most likely explanation for our results: that pike in the LH group had more energy available for growth when they were switched to high food levels because they had reduced their metabolic costs compared to pike from the HL group. Several studies of compensatory growth in fishes has noted that such responses tend to be strongest immediately after being switched from unfavourable to favourable feeding conditions and disappear after a few weeks [27,53]. This raises the intriguing possibility that the time lag before RMRs increase may create a window of opportunity where the scope for compensatory growth is maximized. Whether this represents an adaptive response, or merely a result of metabolic plasticity being a slow process, is an intriguing question for future studies. Taken together, these results highlight the fact that, from a bioenergetic perspective, increases in metabolism can lead to both increases and decreases in growth depending on whether they are also followed by changes in feeding rates.

Our results suggest that the feeding capacity of young-of-the-year pike are mechanistically decoupled from their RMR, and does not change substantially after a five-week period of feast or famine. Specifically, we found that the mean food consumption hovered around 22% of an individual's body mass per week at high food levels regardless of their earlier feeding history (figure 3). In fact, additional observations performed on a subset of individuals at the end of the experiment showed that they did not increase their food intake above this level even when offered an even higher food ration, 37.5% of their wet weight per week, for six weeks (data not shown). It should be noted that the lower intake on week 6 at high food levels mainly reflect that they were only fed three times this week, rather than four, which means that we cannot exclude the possibility that they experienced a brief reduction in their maximum feeding capacity immediately after being switched. Taken together, these observations suggest that young-of-the year pike remain at or very close to their maximal feeding capacity regardless of whether they have up- or downregulated their metabolism. This is in line with earlier observations showing that piscine predators such as pike typically maintain an excess digestive capacity that enables them to eat 2–3 times more than the feeding rates they typically achieve in nature, which appears to represent an adaptation for taking advantage of highly variable and unpredictable feeding opportunities [54]. Studying metabolic plasticity in species that differ in foraging mode and feeding frequency may thus provide important insights into the relationship between metabolism, feeding rate and growth.

However, why do pike, like most species studied to date, upregulate their metabolism when resources are abundant if it does not—by itself—promote faster growth at high food levels? We suggest that the answer may lie in how changes in RMR influence individual foraging success at high prey densities under natural conditions. In a laboratory experiment such as ours, individuals can detect and catch prey nearly instantaneously after being fed. As a result, traits such as cruise speed, burst speed or burst duration have limited scope for influencing the amount of food individuals can eat. However, under natural conditions, even when prey abundance is high, traits such as these may have a considerable impact on a predator's prey encounter rate and capture probability, and these traits may, in turn, be strongly influenced by an individual's metabolic state. Whether increases and decreases in RMR on the scale observed here influence predator behaviours and functional response parameters under naturally low, heterogeneous and unpredictable prey densities remains a fruitful avenue for future research.

## Data Availability

Data and R code for this study are accessible via the electronic supplementary material (electronic supplementary material, files S1–S3). The data are provided in electronic supplementary material [55].
